# Toward Culturally Responsive Algebra: Evaluating and Revising Mathematical Tasks

**DOI:** 10.1007/s10763-026-10667-x

**Published:** 2026-04-27

**Authors:** Hyunyi Jung, Chonika Coleman-King, Taryrn T. C. Brown, Frances Harper, John Grab, Takeshia Pierre, Ji-Won Son, Jiyeong Yi

**Affiliations:** 1 College of Education and Human Development, Texas A&M University, College Station, TX USA; 2 College of Education, University of Florida, Gainesville, FL USA; 3 Theory & Practice in Teacher Education, University of Tennessee, Knoxville, TN USA; 4 New York, NY USA; 5 Department of Education, Tufts University, Medford, MA USA; 6 University at Buffalo, New York, NY USA; 7 Iowa State University, Ames, IA USA

**Keywords:** Algebra, Culturally responsive mathematics curriculum, Curriculum design, Social justice

## Abstract

Persistent inequities in mathematics education point to a critical need to reimagine curricular tasks to better support marginalized student groups. While culturally responsive pedagogy has received growing attention, mainstream algebra curricula still largely fail to reflect students’ lived experiences or sociopolitical realities in meaningful ways. This study evaluates two units from a widely used algebra curriculum, synthesizes conceptual frameworks for developing culturally relevant materials, and reimagines traditional math tasks to better reflect the experiences of Black and Latinx students. An analysis of 50 original tasks revealed a consistent focus on academic rigor, yet showed minimal evidence of cultural relevance, representation, or integration of sociopolitical and social justice themes. In response, we revised selected tasks to preserve – or enhance – their academic rigor while incorporating elements of cultural competency and social justice. This work recognizes the complexity of such transformations and the potential challenges involved. By sharing the framework, evaluation process, and examples of both original and revised tasks, this study offers practical insights for educators and curriculum developers striving to support both equity and excellence in mathematics education.

## Introduction

In mathematics education, mathematics tasks wield immense influence on students’ mathematics learning opportunities. Even though teachers may take up mathematics tasks in different ways, instructional plans largely align with the characteristics of curriculum materials (Choppin et al., 2021). Thus, learning opportunities are facilitated by how mathematics is framed through tasks, and promoting equity and justice within and through mathematics necessitates consideration of how mathematics tasks support such an emphasis. In other words, to achieve equity goals, tasks must extend the framing of mathematics to encompass an awareness of the social justice or injustice that permeates our world. To illustrate, consider a mathematics curriculum that incorporates two real-world problems involving the multiplication of decimals: one related to purchasing candy and the other centered on factory workers enduring significantly low minimum wages (Gutstein & Peterson, 2005). Depending on which problem a teacher selects, students may engage in mathematics either to compute the cost of candy or to critically examine the prevailing social order and societal injustices. Consequently, the task shapes students’ disposition toward mathematics – determining who it is for and what purposes it serves (Aguirre et al., 2013a) – in this case either serving as a tool of consumerism or as a tool for labor justice.

Scholars have established that mathematics, and especially school mathematics, is inherently political and grounded in specific social and cultural contexts (e.g., Battey & Leyva, 2016; Bishop, 1988; Gutiérrez, 2010/2013; Skovsmose, 2020). Drawing inspiration from critical race theory (Delgado & Stefancic, 2001; Ladson-Billings, 2009a) and culturally relevant pedagogy (Ladson-Billings, 2014) makes evident how the curriculum within public schools in the United States plays a central role in either perpetuating students' limited awareness of existing societal inequalities or fostering their consciousness of these issues. Scholars have sounded the alarm that a purportedly ‘neutral’ curriculum inadvertently perpetuates a white supremacist master narrative (Ladson-Billings, 2009a), thereby racializing mathematics learning by entwining mathematical experiences with white cultural norms and ideology (Battey & Leyva, 2016; Bullock, 2017; Leyva et al., 2022; Martin, 2006). In light of this sobering reality, the exigency arises to reshape curricula, halt the cycle of systemic racism, and empower students to recognize and challenge social inequalities. Notably, school curricula frequently omit the lived realities of students marginalized in mathematics on the basis of race and/or ethnicity (i.e., students of color), necessitating the integration of their lived realities into mathematics tasks. Curricular materials provide a lever for changing which mathematics is taught, how it is taught, and what purposes it serves (Rezat et al., 2021). Thus, inclusion of culturally, socially, and politically relevant dimensions in the mathematics curriculum possesses the potential to empower both students of color and their educators to challenge systemic racism, ultimately reframing mathematics education as a liberating practice rooted in transformative educational approaches (Buenrostro & Radinsky, 2020; Harper & Kudaisi, 2023; Kokka, 2025; Leonard et al., 2010). An emphasis on reshaping curricular materials may support students in recognizing how mathematics learning, as constructed in mainstream schools, is applicable to their lives in ways that might benefit them and their communities.

While a neutral curriculum unwittingly upholds systemic racism by marginalizing specific racial and ethnic groups, the adoption of a sociopolitical perspective in mathematics education equips curriculum developers and educators to confront intricate questions involving identity, agency, power, and the sociocultural and political dimensions that envelop mathematics, learning, and instruction (Gutiérrez, 2010/2013). Embracing a sociopolitical and culturally relevant approach to (re)designing mathematics curriculum enables the recognition of mathematics as a product of our shared human experience and history. Mathematics tasks, as an essential component of curricular materials, wield profound significance due to their pivotal role in shaping the perceptions and knowledge of both students and educators, but the processes of reforming curriculum are rarely described in detail within mathematics education scholarship (Rezat et al., 2021). We, therefore, elaborate on the process our research team devised to evaluate a mainstream and ‘race-neutral’ curriculum and to reshape the curriculum by revising algebra tasks to promote cultural responsiveness for Black and Latinx students in the United States. Accordingly, this article responds to an urgent call for research dedicated to providing insights on the systematic design of mathematics tasks that resonate with the cultural contexts of students of color (Gallivan, 2017). Objectives of this project included (a) the construction of a conceptual framework informed by pertinent literature for the evaluation and development of culturally responsive curricular materials; (b) the evaluation of cultural responsiveness within a mainstream algebra curriculum, and (c) the transformation of mainstream mathematics tasks within an online algebra curriculum into culturally responsive challenges tailored to the unique experiences and needs of Black and Latinx students in the United States. For this paper, we aimed to answer the following research question: How can the application of conceptual frameworks derived from relevant literature on culturally responsive curriculum aid in evaluating original mathematics tasks and gauge the extent to which revised tasks align with features of culturally responsive mathematics?

## Tools for Evaluating Mathematics Curriculum and Instruction

A number of research tools exist that allow for the study of various aspects of mathematics teaching and learning, and those tools can provide practitioners, including teachers and curriculum developers, with a framework to support instructional change (Boston et al., 2015; Smith, 2014). Most of these research tools serve as observation protocols, which typically include a range of dimensions scored on descriptive rubrics to differentiate the quality or frequency of observable activities and behaviors. For example, one such widely used protocol, the Instructional Quality Assessment for Mathematics (IQA) instrument, focuses on the adoption of reformed practices in mathematics. Teaching is scored on a 4-point scale to assess the entire lesson for academic rigor (i.e., cognitive demand) and mathematical quality of classroom discourse (i.e., accountability talk). Observers determine the level of rigor on the basis of a rubric and accountability talk on the basis of frequency and level of observed behavior (Boston, 2012). Other protocols used in mathematics, such as the Mathematical Quality of Instruction (MQI; Charalambous & Litke, 2018; Learning Mathematics for Teaching Project, 2011), and Teaching for Robust Understanding in Mathematics (TRU Math; Schoenfeld, 2014, 2020; Schoenfeld et al., 2014) instruments, also focus on the richness of disciplinary discourse and the rigor or extent of engagement in mathematics. Such dimensions capture broader instructional trends toward student sense making, which is an important yet insufficient aspect of culturally responsive mathematics education (Gutiérrez, 2011; Ladson-Billings, 2009b). Moreover, the aspects of teaching in most need of improvement for cultural responsiveness have proven difficult to assess and develop using such protocols (Gitomer et al., 2021).

Other scholars have prioritized the development of observation protocols for culturally responsive teaching and learning, such as the Culturally Responsive Instruction Observation Protocol (CRIOP) (Powell et al., 2016). This protocol assesses culturally responsive instruction, which explicitly seeks to foreground the sociocultural and sociopolitical experiences of students of color and to develop critical consciousness alongside academic competencies. The protocol operationalizes culturally responsive instruction holistically, using (1) foundational elements (teacher care, family collaborations, classroom relationships); (2) pedagogical elements (assessment, planned experiences, pedagogy); and (3) critical elements (instructional conversation, socio-political consciousness) (Powell et al., 2016). In mathematics education, Aguirre and del Rosario Zavala (2013) recognized the need to develop a professional development resource that assists teachers in developing competencies related to culturally responsive mathematics teaching. Aguirre and Zavala used data from a three-year professional learning program to create an observation tool focused on eight key dimensions around cognitive support, depth of student understanding, students examining mathematics, mathematical discourse, student engagement, support for academic language, scaffolding for English learners, funds of knowledge, and social justice. This culturally responsive mathematics teaching tool fostered critical analysis of mathematics lessons and pedagogical dialogue among teachers.

Such observation protocols provide a tool for evaluating the implementation of curriculum and instruction in mathematics classrooms, but they offer limited guidance for assessing how the curriculum itself aligns with features of culturally responsive mathematics. This gap is important because instructional plans largely align with the features of curriculum materials, which subsequently can shape the learning opportunities of students (Choppin et al., 2021). When research tools focus attention on curricular materials themselves, the complex relationship among mathematics tasks, teaching and learning emerges more clearly. For example, research (e.g., Stein et al., 1996) using the IQA protocol, which includes a rubric for assessing cognitive demand of mathematics tasks as written, suggests high-level cognitive demand tasks are necessary for teaching and learning mathematics in cognitively demanding ways. In other words, when instruction builds from low-level cognitive demand tasks, students almost never have opportunities for cognitively demanding mathematics engagement. Instead, teachers are more likely to lower the cognitive-demand of tasks during the set up (Stein et al., 1996). The implications of such research suggest that researchers and practitioners need tools for assessing the cultural responsiveness of written tasks, separate from implementation. Such tools can support the redesign of mathematics curricular materials for cultural responsiveness, as a necessary starting point for instructional planning and implementation.

## Conceptual Framework Informing the Development and Evaluation of Culturally Responsive Mathematics Tasks

We recognize that many researchers have explored and applied culturally relevant pedagogy (Ladson-Billings, 2009b) and culturally responsive teaching (Gay, 2009) in the development, analysis, and instruction of mathematics lessons. Gay (2009) emphasized that culturally responsive mathematics teachers recognize the cultural resources of diverse students, connect students’ lived experiences to mathematical concepts, and maintain high cognitive demand while providing appropriate support. Moreover, Ladson-Billings’s (2009b) research on successful teachers of African American students provided the foundation for culturally relevant pedagogy, which ensures students’ academic achievement, fosters their cultural competence, and supports development of their sociopolitical consciousness. Taken together, these two bodies of work provide guidance for (re)designing culturally responsive curriculum. Culturally responsive curriculum can be thought of as the incorporation of content that is meaningful and relevant to students of color, including information about their histories, cultural backgrounds, lived experiences, and perspectives to foster cultural competence (Ladson-Billings, 2009b).

In creating a rubric for evaluating and revising existing algebra tasks to become culturally responsive algebra tasks for Black and Latinx students, we used the following bodies of literature to build from the foundation work of Gay and Ladson-Billings: funds of knowledge (to include sources within and beyond school), teaching mathematics for social justice (to develop sociopolitical consciousness), representations of marginalized groups (to foster cultural competence) and cognitive demand of mathematical tasks (to support academic achievement). We acknowledge that our tool does not encompass all the dimensions and emphases identified in prior work (cf. Aguirre & del Rosario Zavala, 2013), as this study focused specifically on task revision rather than lesson implementation. While we recognize the importance of developing tools that support teachers’ competences in culturally responsive mathematics teaching in their daily practices, we argue that a tool targeting the design and revision of mathematical tasks, rather than their implementation, also holds significant value. Instructional plans are often shaped substantially by the features of curriculum materials (Choppin et al., 2021), and we hope that a tool designed to guide curriculum materials toward being more culturally responsive to Black and Latinx students can serve as one of multiple approaches that contribute to raising broader awareness around curriculum development. Table 1, presented below, illustrates the synthesis of relevant literature around culturally responsive and relevant mathematics curriculum and instruction (e.g., Aguirre & del Rosario Zavala, 2013; Berry et al., 2020; Gay, 2002; Gutstein, 2003; Ladson-Billings, 2014; Larnell et al., 2017; Peoples et al., 2021; Rubel, 2017; Smith et al., 2004; Yeh, 2021), along with our conceptualization of domains that encompass four aspects: (a) funds of knowledge and cultural competency, (b) sociopolitical and social justice issues, (c) representation, and (d) high academic expectation. Following Table 1, we describe how each domain is grounded in prior research.

**Table 1:** A framework for culturally responsive mathematics tasks based on literature

Domain	Features of the Culturally Responsive Mathematics Task
A: Funds of knowledge and cultural competency	A1: The task provides opportunities for students to incorporate their home and community experiences
	A2: The task allows students to see the value of minoritized students' cultural practices and experiences
	A3: The task affirms varied practices across students' everyday lives and connects them with mathematical contents and practices
B: Sociopolitical and social justice issues	B1: The task provides avenues for students to view mathematics as a means to explore human rights (e.g., the right to life, freedom from slavery, freedom of expression)
	B2: The task allows students to consider the fair distribution of resources and opportunities in society
	B3: The task elevates students' sense of agency in creating positive, equitable change in the society
C: Representation	C1: The task includes pictures or scenarios that resonate with historically marginalized identities (e.g., racial and ethnic minorities, women, individuals with disabilities, working class communities)
	C2: The task acknowledges the origins and history of mathematics within marginalized identities
	C3: The task elevates diverse forms of communication to facilitate students’ understanding of mathematical language
D: High academic expectation	D1: The task requires an understanding of procedures with connections to mathematical ideas and meanings
	D2: The task allows students to consider or use multiple representations (e.g., visual diagrams, graphs, symbols)
	D3: The task invites students to identify their own strategies and examine possible solutions
	D4: The task provides avenues for students to articulate and justify their mathematical ideas

## Funds of Knowledge and Cultural Competency Domain

The Funds of Knowledge and Cultural Competency domain was developed by drawing upon relevant literature that discusses the importance of bringing experiences of students’ homes and community, seeing the value of minoritized students’ cultural practices and experiences, and affirming varied practices across students’ everyday lives and connect them with mathematical content and practices (Aguirre & del Rosario Zavala, 2013; Civil, 2016; Gallivan, 2017; Gay, 2002; Gonzalez et al., 2005; Ladson-Billings, 2014). In recent decades, mathematics research has incorporated ethnographic data to document sources of mathematical practice and problem-solving that occur in the homes of diverse students. For instance, researchers such as González and colleagues (2001) visited diverse communities to understand how funds of knowledge influenced mathematics learning. Students’ funds of knowledge are defined as “the culturally developed knowledge, skills, and experiences of children in their homes, with their families, and in the community (González et al., 1995; Moll et al., 1992, p. 94). Stoehr and colleagues (2022) suggest that there are positive educational outcomes for historically underrepresented students, such as Black and Latinx learners, when funds of knowledge are applied to mathematics instruction. Civil and Quintos (2022) have also positioned cultural and linguistic funds of knowledge as intellectual resources, where community practices, skills, and experiences were viewed as resourcefully rich to mathematics education.

In this 2001 study, Gonzalez and colleagues described the connection of funds of knowledge and mathematics through sewing circles led by Latinx mothers. Authors contended that these mothers, similar to students in classrooms, were not positioned as “consumers of knowledge, but producers of mathematical practices” (González et al., 2001, p. 130). There have been continued efforts to bridge home and community practices with classroom learning and showcased intentionality in providing an explicit focus on mathematics (Civil, 2016), where researchers followed the experiences of families in their everyday lives through household visits and developed modules from their findings. As of recent, researchers have leveraged collaborations with parents alongside their children, finding that their mathematical funds of knowledge helped to provide insight into engaging lessons for students and can directly support and influence students’ mathematical learning (Stoehr et al., 2022; Williams et al., 2020). In addition, focusing on students’ mathematical funds of knowledge has proven beneficial to teachers of diverse learners, where it invites meaningful connections with students and their parents, and is crucial to the bridge-building process between home and school knowledge production (Aguirre et al., 2013b; Turner & Drake, 2016). While much literature has been devoted to Latinx communities, additional research is needed to leverage the funds of knowledge of Black and Latinx students in mathematics. This study helps address this gap in literature, with previous studies serving as foundational to the production of inclusive mathematics instruction developed from the funds of knowledge of Black and Latinx students.

Our goal was to ensure that the written mathematics tasks designed within this domain would enable students to incorporate and reflect upon their experiences from their homes and communities. Moreover, we aimed to create tasks that would allow students to recognize the value of cultural practices and experiences from minoritized groups. Additionally, these tasks were designed to acknowledge and affirm the diverse practices that students experience in their everyday lives and establish connections between these practices and mathematical concepts and methods. Through our collaboration in revising the tasks, we emphasized the importance of authentic, real-world applications of mathematics that reflect how mathematics is genuinely used in various cultural contexts, including at home and in the community. This involved a shift from typical word problems to authentic problems that are more likely to be encountered in society. While our goal was to broaden representation in mathematics curriculum, we also recognized that racial and ethnic groups are not a monolith and tried to account for the variation we saw in our participants whose experiences varied based on geographic location, immigrant background, gender, and social class background, to name a few.

## Sociopolitical and Social Justice Domain

The initial Sociopolitical and Social Justice framework was thoughtfully crafted to acknowledge and respect the valuable contributions of other researchers in the field who emphasized the importance of considering social justice into mathematics teaching and learning (e.g., Berry et al., 2020; Larnell et al., 2017). Mathematics has been highlighted as a tool that can promote social justice in education – where enacting more socially just mathematics can help to disrupt the marginalization of Black students. Examples of this disruption have emerged in mathematics education literature, particularly in the interrogation of whiteness in mathematics curriculum (Harper et al., 2021), incorporating mathematics that highlights important cultural topics such as Black ancestry and relative (counter)stories throughout Black history (Leonard, 2020), and developing methods for educators to implement more socially just practices and curriculum (e.g., Bartell, 2013; Harper & Kudaisi, 2023; Jung & Magiera, 2023; Jung & Wickstrom, 2023; Wickstrom et al., 2024; Yi et al., 2026). In addition, while some studies have focused on the experiences of mathematics teachers teaching for social justice with implications for future practice (Raygoza, 2020), some scholars have extended conversations relative to diverse student experiences. For example, Jung and Brand (2021) described how African American middle school students approached social justice issues such as homelessness, which resulted in fruitful discussions among students, in addition to developing plans for action to solve these problems through mathematical tasks (e.g., strategic purchasing and budgeting while considering optimization and bringing home practices). This lesson served as an example of what authors consider the five steps to student actions when engaging in a social justice mathematics task, which include: (a) analyzing the issue at hand along with its associated social justice implications; (b) strategizing methods for tackling the social justice concern; (c) transforming the scenario with mathematical lens; (d) ensuring the viability of the solution given the contextual limitations; and (e) implementing the solution to the context (Jung & Brand, 2021).

Furthermore, though literature has mainly focused on the incorporation of socially just practices in mathematics classrooms and improving teacher and student outcomes through these practices, others have investigated and problematized common findings in mathematics social justice literature. Harper (2019) conducted a meta-synthesis of 35 qualitative articles that showcased social justice in mathematics classrooms. This study highlighted the importance of: (a) addressing race in social justice mathematics topics and how it allowed for centering the voices of minoritized students, (b) critiquing liberal views that disguise subtle forms of racism, and (c) the promotion of rigorous mathematical work. Literature on teaching mathematics for social justice offers a foundation for the ethical and intentional approach of this study.

In the process of revising this framework, we took great care to ensure that the tasks created within it support students in viewing mathematics as a tool for understanding and advocating human rights, including fundamental rights such as the right to life, freedom from slavery, environmental justice, and freedom of expression. We also addressed social justice concerns by designing tasks that encourage students to contemplate the equitable distribution of resources and opportunities within society. Furthermore, these tasks were specifically crafted to empower students by fostering their capacity to act, enabling meaningful contributions to positive and equitable change in society.

## Representation Domain

Our initial Representation Framework was built on prior work (e.g., Hunter & Miller, 2020; Peoples et al., 2021; Yeh, 2021). In a scoping review of thirty studies, Hunter and Miller (2020) found that approaches to embedding culture into mathematics lessons involved not only contemporary examples from daily life, such as those discussed above in the Funds of Knowledge and Cultural Competency Domain, but also included Indigenous mathematics concepts and culturally diverse mathematics language and symbols. The incorporation of mathematics in its non-Western cultural context has shown to increase the mathematics achievement of students (Lipka & Adams, 2004). These foundational works have provided insights into the effective representation of mathematical concepts, especially in relation to historically marginalized groups such as racial and ethnic minorities, women, individuals with disabilities, working class communities. Researchers and teacher educators have designed tasks that incorporate scenarios relevant to historically marginalized groups and that elevate diverse forms of communication (e.g., Carlisle et al., 2025; Sutcliffe et al., 2025; Yi et al., 2025; Zhang et al., 2025). In designing our tasks, we acknowledged the origins and historical contributions of mathematics within marginalized communities. We also endeavored to embrace diverse forms of communication to make mathematical language more accessible. This entails not only utilizing mathematical symbols and notation but also incorporating alternative modes of expression, such as storytelling, visual arts, and real-world applications related to mathematics. By diversifying the means through which mathematical concepts are presented, we aimed to address the diverse learning needs and strengths of students.

## High Academic Expectation Domain

The initial High Academic Expectation framework was developed based on existing literature that centers around the concept of academic success for all students and the use of mathematical task analysis guides that emphasize high cognitive demand (e.g., Gallivan, 2017; Ladson-Billings, 2014; National Council of Teachers of Mathematics [NCTM], 2014; Rubel, 2017; Smith et al., 2004). Literature suggests that U.S. teachers find it challenging to offer their students mathematics tasks that demand a high level of cognitive demand (e.g., Agterberg et al., 2022). Designing tasks or modifying existing tasks is not a straightforward process for teachers. When prospective teachers are asked to enhance cognitive demand of tasks, they do not always succeed, and the contextual features they add are sometimes unreasonable (Lee et al., 2024). This concern is especially relevant in mathematics classrooms, where these particular tasks consist of completing a single or a set of complex problems that focuses students’ focus toward a specific mathematical idea (Stein et al., 1996). While researchers have examined cognitive demand tasks in various international contexts (e.g., Deepak et al., 2023; Ni et al., 2018), there has been a limited emphasis on mathematical tasks designed for racially marginalized students. This paper contributes to the existing literature by addressing the gap and presenting cognitively demanding mathematical tasks tailored to benefit all students, including Black and Latinx students.

Drawing from the foundation laid by Smith and colleagues (2004), we ensured that the tasks within this framework require a deep understanding of mathematical procedures while fostering connections to broader mathematical concepts and meanings. These tasks also offer students the opportunity to explore and utilize multiple representations, including visual diagrams, graphs, and symbols. Moreover, the tasks incorporated into the High Academic Expectation framework encourage students to identify and employ their own problem-solving strategies and to critically examine potential solutions. This approach not only supports students in reasoning and justifying their mathematical ideas but also provides avenues for their growth in the subject.

## Methods: Our Attempt to “Balance the Equation”

Our emphasis on using culturally responsive frameworks to evaluate and revise tasks builds from a larger attempt to address what the Gates Foundation describes as a “Global Grand Challenge”– an attempt to “Balance the Equation” by ensuring exposure to equitable Algebra 1 learning experiences to help provide more robust opportunities for Black and Latinx youth. In this project, funded by the Bill and Melinda Gates Foundation, examining the literature was our first step in attempting to address inequities in algebra. The development of the above frameworks (Table 1) occurred in tandem with a series of interviews conducted with Black and Latinx middle and high school students and their parents to find out from them what elements of their culture, values, and experiences they wanted to see represented in a mathematics curriculum. Drawing from interview themes in tandem with the application of our above research-based frameworks, we revised several units of a widely used online mathematics curriculum. In this way, both the research base in mathematics education and the voices of Black and Latinx youth and families guided the curricular redesign, which reflects a promising approach for maximizing the potential for curricular materials to serve as instruments of change (Rezat et al., 2021). We revised approximately fourteen existing lessons (which included fifty written mathematics tasks) to make them culturally responsive to Black and Latinx students.

## Curriculum and Task Context

For this project, we collaborated with Math Nation and K-Algebra 2 | Interactive Online Math Program (2025), a mainstream and widely-used mathematics program, built around a hybrid format combining online and face-to-face learning. A blended approach, increasingly common in mathematics education, supplements traditional classroom instruction with online, self-paced instruction that allows students to revisit and extend mathematical ideas and topics (Borba et al., 2016). The *Math Nation* program enhances the problem-based Illustrative Mathematics | Kendall Hunt (2019) curriculum by incorporating teacher planning guides and implementation tools alongside digital tools for students. Student-facing tools include a social networking wall for peer-to-peer engagement, a tool allowing students to set their own learning goals, and on-demand, instructional videos in English and Spanish.

In our partnership with *Math Nation*, we sought to revise a select number of lessons towards a prototype for *Culturally Responsive Math Nation* as we prepared for a classroom-based pilot. Our work to redesign curricular materials from *Math Nation* involved redesigning written mathematics tasks from the *Illustrative Mathematics* Algebra I curriculum and creating new online supports, such as new English and Spanish videos for the redesigned lessons. With our partner, we selected fourteen lessons (which included fifty written mathematics tasks) from across two units for the prototype. More specifically, we chose four lessons from Unit 3: Two-variable Statistics and ten lessons from Unit 4: Functions for evaluation and revision, as a starting point for curriculum redesign. Lessons from Unit 3 focused on linear models and correlation; and lessons from Unit 4 included topics related to interpreting and using graphs and function notation to describe rules, find average rates of change, and make comparisons.

## Team-Led Lesson Evaluation and Revision Process

Once the first author proposed the frameworks (Table 1) synthesized from the relevant literature, the project team collaboratively met to discuss the frameworks. The team then practiced applying the frameworks by evaluating and revising sample lessons together. Next, the team divided the revision tasks, with small sub-teams of two or three team members, convening individually and collectively to review and amend the tasks. Each sub-team member employed the frameworks (Table 1) to individually evaluate the original tasks in each lesson before collaboratively comparing their evaluation results and reaching a consensus. Then, they proceeded to revise the tasks accordingly, using the frameworks as a guideline for improving the cultural responsiveness of the written tasks.

Once all team members completed the task revisions, the first and fifth authors conducted a comprehensive review of all the lessons. The unit of analysis was a mathematical task under each lesson, which included three to four tasks. Specifically, these authors individually coded each task based on the frameworks and subsequently met to address any discrepancies. To establish inter-rater reliability, the two authors randomly selected five original tasks and five revised tasks and individually coded them using the four domains. The inter-rater reliability of codes for each domain was assessed and is presented in Table 2 below before discussing any disagreements.

**Table 2:** Inter-rater Reliability of Codes from Each Domain

	Domain A: Cultural Competency	Domain B: Social Justice	Domain C: Representation	Domain D: High Academic Expectation
Inter-rater reliability	87%	93.3%	73.3%	85%

After reaching a consensus on coding, the two authors individually analyzed the rest of the tasks and met to resolve further discrepancies until reaching a consensus. The final version of this analysis is shown in Table 3 included in the Findings section. Furthermore, the authors conducted a detailed comparison of tasks, focusing on their alignment with the four key domains. Then, the first and seventh authors selected and summarized sample tasks to exemplify each of these domains and the characteristics of the original and revised tasks. This comparison highlights the essence of the revision process in relation to these four domains and relevant features, which we describe in the Findings section.

**Table 3:** Occurrences of the Features of Culturally Responsive Mathematical Tasks for Original and Revised Algebra Lessons

	Domain A: Cultural Competency		Domain B: Social Justice		Domain C: Representation		Domain D: High Expectation	
Content (Lessons)	Original	Revised	Original	Revised	Original	Revised	Original	Revised
Linear Models and Correlation Coefficient (Unit 3 Lessons 4, 5, and 7, 8)	5	26	0	15	6	19	36	42
Function Notation (Unit 4 Lessons 1–5)	5	17	0	12	2	18	30	54
Function Representations (Unit 4 Lessons 6–10)	3	24	0	11	2	24	45	69
Total	13	67	0	38	10	61	111	165

Each Lesson included three to four tasks

## Student-Centered Lesson Evaluation and Revision Process

Simultaneously with team-led task modifications, we gathered insights from Black and Latinx students, parents and teachers via individual and focus group interviews to elevate their voices in the curricular redesign process. Fifteen students, four parents, and two teachers participated in interviews. Individual interviews with parents and teachers focused on soliciting their general input into the concept of culturally responsive algebra. We asked them to share their perspectives on how their children/students experience algebra learning in culturally (ir)relevant ways, and they shared ideas for more culturally responsive approaches and problem contexts. In 60–90 min focus group interviews, Black and Latinx students joined breakout rooms on Zoom, where we asked them to work through some mathematics tasks revised by the team. Then, students discussed their experiences with these redesigned tasks and answered the interviewer’s questions: (a) How does this problem or activity compare to math activities you typically experienced?; (b) Does this version of the problem reflect your interests or experiences?; (c) What other topics might you suggest we include?; (d) Does this problem help you see mathematics as a means to understand the world and promote positive, equitable change? If so, how? Using a qualitative approach to thematic analysis (Wicks, 2017), we identified considerations for ongoing curricular design. Students’ insights, in particular, informed further redesign of tasks, helping us to refine the tasks to better align with Black and Latinx cultural perspectives and educational experiences. Importantly, student input confirmed which ideas and experiences seemed to resonate with most students and pointed to areas where we needed to anticipate wide variation in Black and Latinx students’ interests and experiences.

## Finalizing Curricular Revisions

Each revised written task went through multiple rounds of review by different team members, who considered the tasks’ alignment with the frameworks and student input, before being finalized for incorporation into the online curricular materials. In an effort to avoid essentializing the experiences of Black and Latinx students, we prioritized incorporating a broader range of options for each student into the final prototype for *Culturally Responsive Math Nation*. The online curricular program allowed us to respond to variation in ways that the redesign of written tasks did not. Specifically, new instructional videos were recorded for each redesigned task and uploaded to the online platform. Students could select instructors representing different social identities (Black man, Black woman, and Latino man), choose the language of instruction (Spanish or English), and access videos in which teachers read and explained the problems and their contexts. The revised curriculum was implemented fully online, with both English- and Spanish-language teachers providing these explanations in the instructional videos. We recognize that the curriculum materials we created do not and cannot reflect all the nuances of Black and Latinx experiences; and they necessarily reflect the ideas of the students, parents and teachers we interviewed, as well as our interpretation of their responses and our own perspectives. We offered up choice in topic selection as a means of availing Black and Latinx students to a number of different kinds of representation of their values, culture, and experiences and to reduce the appearance of essentialism.

## Findings

In this paper, we present findings from the evaluation and redesign of written mathematics tasks aimed at increasing their cultural responsiveness. As we outlined in our methods section, our revisions to written curricular tasks constituted only one part of our overall efforts towards *Culturally Responsive Math Nation*, but consideration of other curricular materials fell outside the scope of this manuscript. Specifically, we sought to understand how the conceptual frameworks derived from relevant literature aided us in evaluating original tasks and then gauging the extent to which their redesign (led by the research and development team and informed by student, parent and teacher input) aligned with features of culturally responsive mathematics. Although limited in scope, our findings make an important contribution to by providing evidence of the *processes* for reforming curriculum with the goal of leveraging curricular materials for change towards culturally responsive mathematics education.

## Evaluation of Tasks

In this section, we begin by providing an overview of the lesson evaluation summary. This is done by displaying the counts of the features of culturally responsive mathematics tasks within each domain for both the original and revised algebra lessons. Subsequently, we offer sample original and revised tasks to describe the characteristics of the original and revised algebra tasks. Table 3 serves as a summary, presenting the counts of the features for both the original and revised algebra lessons across each of the defined domains, namely *cultural competency, social justice, representation,* and *high expectation.* Each column in the table includes the occurrences for specific mathematical content areas, such as Linear Models and Correlation Coefficients, encompassing four or five lessons.

In general, the original lessons have consistently shown higher numbers in the category of Domain D: High Academic Expectations. This means that the original lessons often required an understanding of procedures with connections to mathematical ideas, allowed students to consider or use multiple representations, invited them to identify their own strategies and examine possible solutions, and provided avenues for them to reason and justify their mathematical ideas. When we revised the lessons, our goal was to preserve or enhance High Academic Expectations, and we often achieved this by introducing additional opportunities for students to utilize multiple representations, justify mathematical ideas, and consider multiple solution approaches. We also made efforts to bolster Domain A: Cultural Competency and Domain C: Representation through lesson revisions. This involved integrating concepts about the cultural practices, experiences, and everyday lives of minoritized students, as well as incorporating images and scenarios that relate to historically marginalized identities. Additionally, we expanded the range of communication forms to facilitate students’ understanding of mathematical language. In the original lessons, we did not identify any instances of Domain B: Social Justice. During our revision, we made an effort to design tasks that encourage students to contemplate the equitable distribution of resources and opportunities in society, consider environmental justice issues, and examine unfair social structures. We aimed to foster a great sense of agency in contributing to positive, equitable changes within society (Table 4).

**Table 4:** Occurrences of the Features of Culturally Responsive Mathematical Tasks for Tasks Shown in Figs. 1–8

	Domain A: Cultural Competency		Domain B: Social Justice		Domain C: Representation		Domain D: High Expectation	
Task (Figures)	Original	Revised	Original	Revised	Original	Revised	Original	Revised
Best Fit Line (Figs. 1 & 2)	1	2	0	1	1	2	4	4
Linear Models (Figs. 3 & 4)	0	3	0	2	1	2	3	4
Function Values (Figs. 5 & 6)	0	1	0	2	1	2	1	4
Creating a Graph Using Functions (Figs. 7 & 8)	0	1	0	1	0	1	4	4

**Fig. 1:**
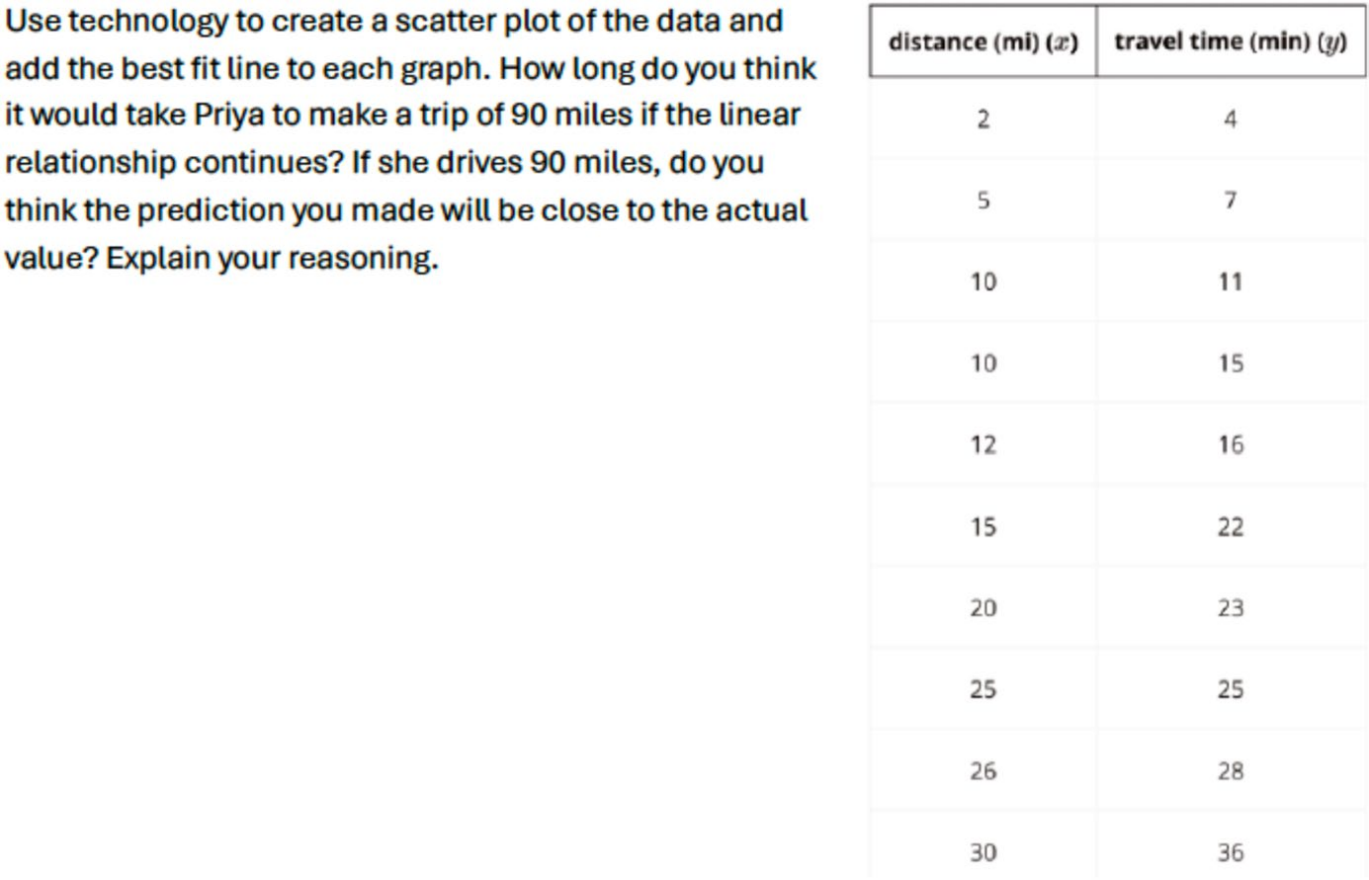
Sample Original Task Focused on the Best Fit Line

**Fig. 2:**
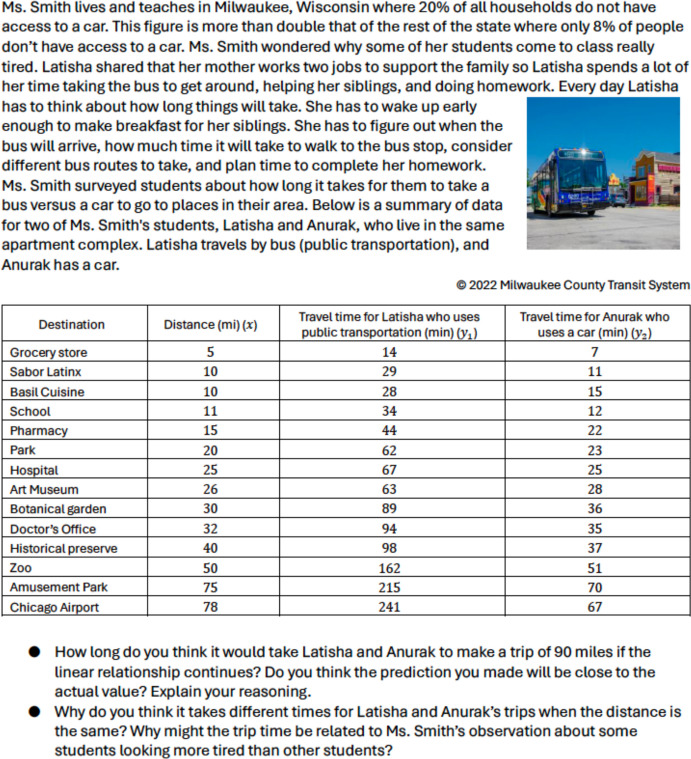
Sample Revised Task Focused on the Best Fit Line

**Fig. 3:**
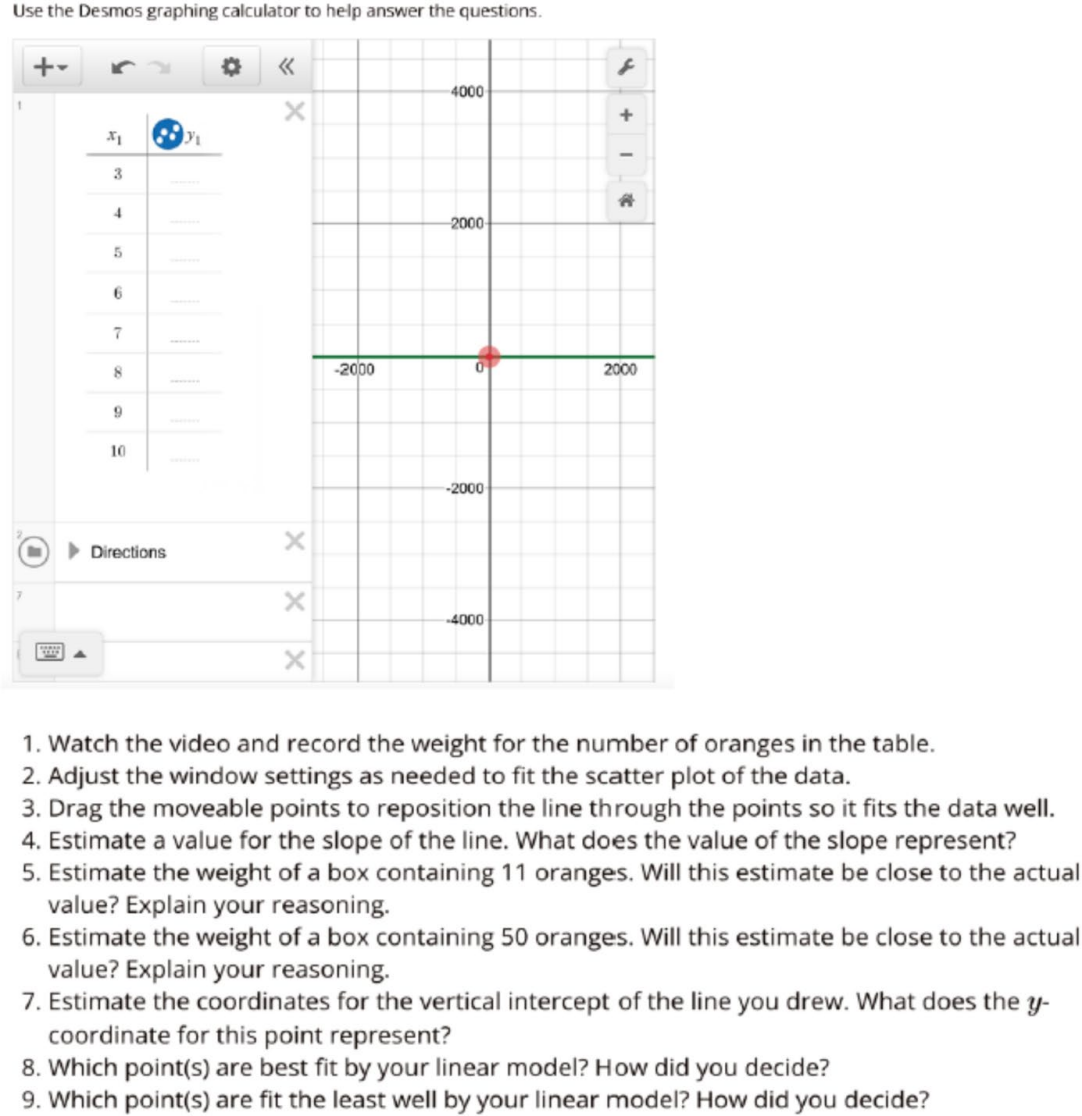
Sample Original Task on Linear Models

**Fig. 4:**
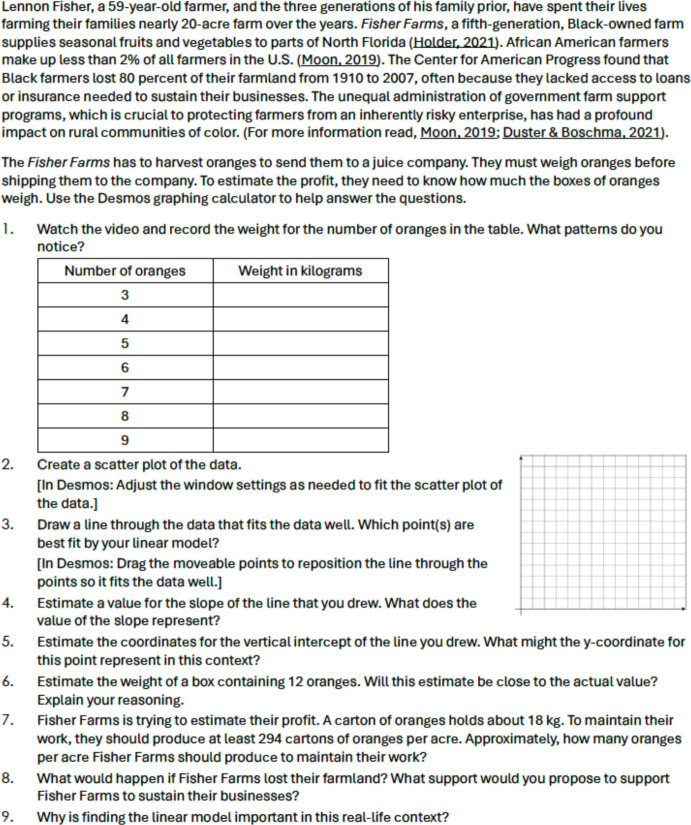
Sample Revised Task on Linear Models

**Fig. 5:**
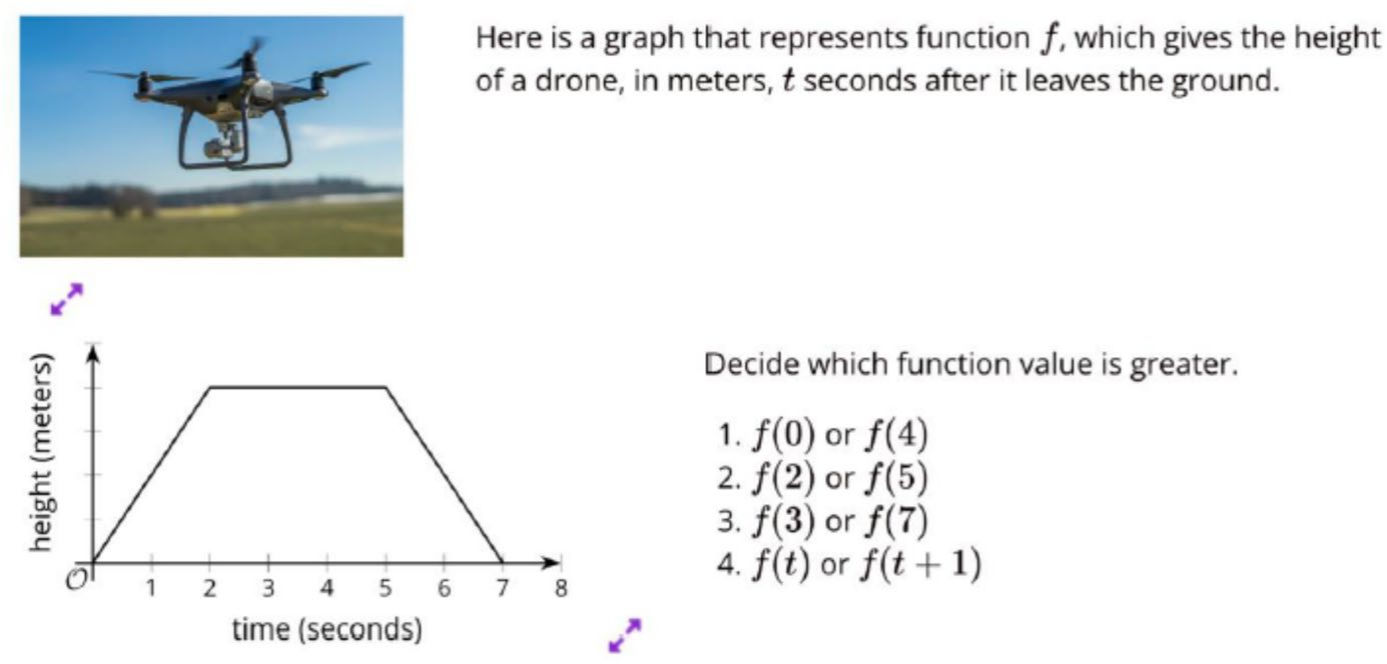
Original Task on Function Values

**Fig. 6:**
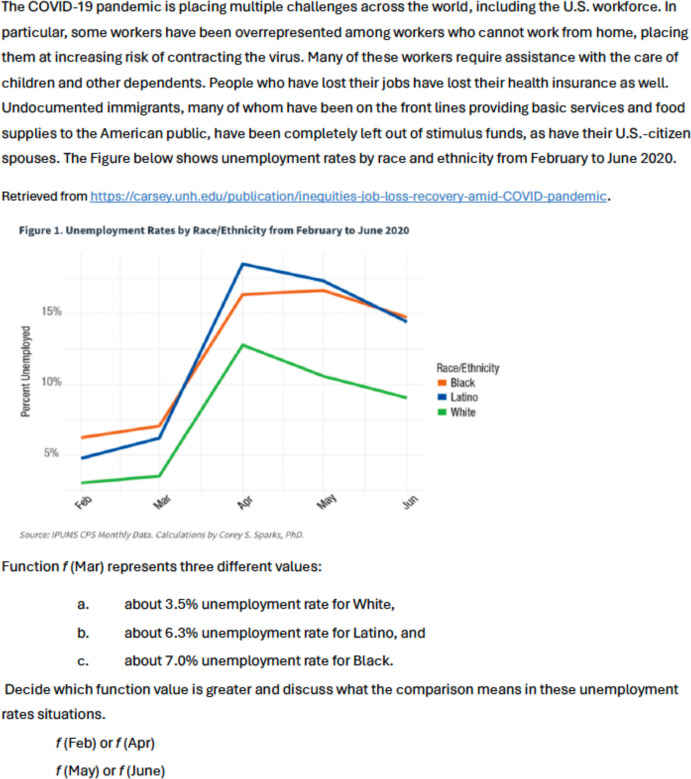
Revised Task on Function Values

**Fig. 7:**
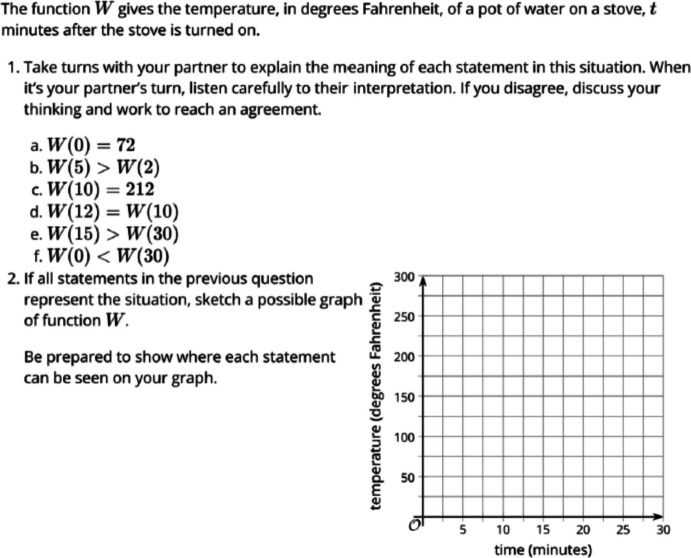
Original Task on Creating a Graph Using Functions

**Fig. 8:**
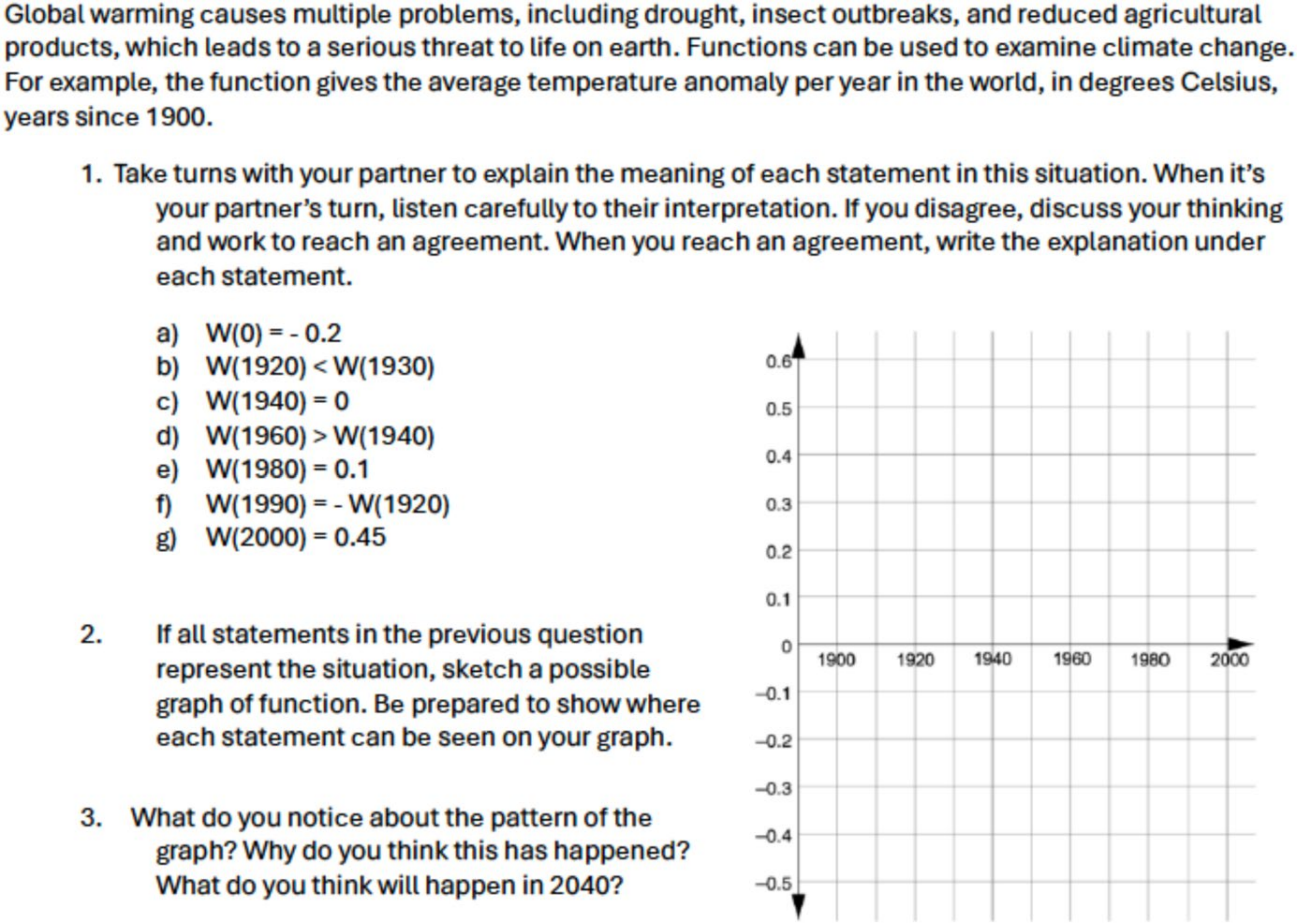
Revised Task on Creating a Graph Using Functions

## Examples of Tasks

To illustrate more details of these changes, we provide samples of original tasks and revised tasks (Figs. 1 through 8) to show the nature of the tasks and how they changed using the framework.

While both tasks affirm a practice across student lives and connect it to mathematical content and practices, the revised task provides a more explicit connection to these practices (See Feature A3 in Table 1). The revised task allows students to connect to their own experiences or understand others’ lives, providing an opportunity to empathize with others (Feature A1 in Table 1). The original task does not contain a social justice context, whereas the revised one incorporates a story that centers on a problem to be solved in the context of justice. The revised task highlights the disparities in transportation access and its impact on students' daily lives (Feature B2), embedding the importance of the fair distribution of resources and opportunities in society. We acknowledge that associating bus use with specific racial groups could risk reinforcing stereotypes, while it also reflects certain social realities, as our tasks were designed to align with students' lived experiences. To avoid promoting a monolithic view of racial groups and perpetuating these stereotypes, further work is needed in designing accompanying teacher guides and task implementations. For example, teachers could emphasize the mathematical reasoning and resources that a student like Latishia might develop through using the bus—opportunities that may differ from students who primarily have access to cars. In addition, acknowledging the systemic opportunity gaps that students like Latishia have historically faced—such as financial challenges—can help prevent the misconception that wealth is solely the result of individual effort or grit. The narrative of Latisha’s experiences emphasizes the additional burdens placed on individuals who lack access to private transportation, contributing to a broader understanding of socioeconomic disparities. In terms of Domain C: Representation, the original task includes a name (Priya) assumed to imply a representation of a historically marginalized group, while the revised task reflects not only names but also the experiences of marginalized groups of students (C1). By surveying students like Latisha and Anurak and comparing their transportation experiences, the task encourages students to analyze and interpret data related to transportation access. This approach not only integrates real-world context into mathematical discussions but also introduces scenarios that might resonate with some historically minoritized groups. Both tasks offer elements of high expectations, including using multiple representations in sense-making and mathematical justification (D1, D2, D3, and D4).

As another example, Figs. 3 and 4 present an original task and the revised task focusing on linear models. Both tasks involve the development of linear models within the context of recording the weight for a varying number of oranges. In the original task, students are tasked with recording weights and estimating values for different quantities of oranges, but the purpose behind these actions is not explicitly addressed. Conversely, the revised task goes beyond the mere mathematical aspect by encouraging students to consider the experiences of the Black community. It highlights how mathematical practices are employed by other communities, including African American farmers in their work (Features A1, A2, and A3 in Table 3). Unlike the original task, the revised version delves into sociopolitical issues by presenting data revealing that African American farmers constitute less than 2% of all farmers in the U.S. and have experienced an 80% loss of farmland from 1910 to 2007, thus addressing social justice concerns (B1 and B2).

Furthermore, the original task lacks representation of minoritized identities and their mathematical practices, while the revised task includes scenarios that resonate specifically with the Black farming community (C1 and C3). Both tasks facilitate the use of multiple mathematical representations and encourage students to articulate their strategies (D2 and D4). Additionally, they provide avenues for students to articulate and justify their mathematical ideas, fostering a high expectation for a meaningful engagement with the ideas (D4)*.*

Figures 5 and 6 show another set of the original and revised lesson which focuses on function values. Both tasks involve the examination of a graph depicting function *f* in context to determine the greater function value. The original task, centered around observing a drone, may not resonate with all students’ experiences. In contrast, the revised task, focused on COVID-19, aims to be inclusive of all students’ experiences, which include both mainstream and minoritized groups (Feature A1 in Table 1). In Domain B, the original task lacks engagement with sociopolitical or social justice issues. However, the revised task provides opportunities for learners to view mathematics as a means to comprehend societal disparities (B1 and B2).

The graph presented in the revised task is designed to resonate with diverse populations, particularly those who have historically and are recently marginalized during the COVID-19 pandemic (C1). Both tasks encourage students to explore multiple representations, including graphs and symbols, fostering a high expectation for a meaningful understanding of functional representations (D2). The revised task added several features: connecting procedures to their mathematical meanings (D1), encouraging students to generate their own solution methods (D3) and having them justify their mathematical ideas through discussion (D4).

The task, initially centered on function values, is succeeded by a related task that involves creating a graph using functions. Figures 7 and 8 depict the original and revised tasks pertaining to this topic. The original task involves depicting the temperature of water on a stove over time as a function. In contrast, the revised task delves into the issue of global warming, by mathematically representing how it might affect an average temperature which affects the real lives of individuals (A3). While the original task lacks sociopolitical or social justice dimensions, the revised task opens avenues for students to perceive mathematics as a means for comprehending global warming, thereby addressing environmental justice concerns (B1). Both tasks promote diverse forms of communication, such as partner discussions to reach conclusions and providing graph paper, making mathematical language more accessible to students (C3). Additionally, both tasks allow students to employ various representations, including graphs and symbols (D2). They encourage students to identify their own strategies, explore potential solutions (D3), and offer opportunities for reasoning and justifying their mathematical ideas (D4), maintaining a high expectation for student engagement and understanding (*high expectation*). In summary, we acknowledge that there are multiple approaches and diverse extent to which one could consider to revise neutral tasks, and we hope some of the examples we share above would inform others who are interested in similar work for their teaching, curriculum design, and/or research.

## Limitations and Recommendations

Although we went through multiple iterations of collaborative lesson modifications, evaluation, and refinement, we recognize that there is still room for improvement. For example, the use of naming conventions or broad social themes could unintentionally reinforce stereotypes or oversimplify cultural identities. Additionally, linguistic factors may influence how students interpret and engage with problem contexts. While the revised curriculum was implemented as an online curriculum in which both English- and Spanish-language teachers read and explained the problems and their contexts in the instructional videos to support accessibility for students with different language backgrounds, language differences may still shape students’ understanding and interaction with the materials. Future studies could focus on developing tasks that reflect the specific and nuanced experiences of diverse cultural groups, involving additional students and community members in the design process to ensure authenticity and inclusivity, as well as exploring how language supports can be integrated into culturally relevant mathematics tasks to better support linguistically diverse learners.

We also acknowledge that our study does not encompass the full scope of prior literature. Specifically, because our focus was on the development and evaluation of lessons rather than their classroom implementation, certain dimensions of culturally responsive mathematics teaching tools—such as mathematical discourse and student engagement (Aguirre & del Rosario Zavala, 2013), which require enactment in classroom settings—were not addressed. Future studies could investigate how the revised tasks function in practice, examining their effectiveness in supporting students’ mathematical thinking, providing intellectual support, fostering mathematical discourse and communication, promoting active student engagement, and supporting the development of student power, agency, and action. Implementing the refined tasks through classroom piloting—incorporating localized narratives, investigating inter-domain relationships within the framework in practice, and documenting teachers’ decision process—could further enhance cultural specificity and foreground culturally responsive mathematics pedagogy.

## Conclusion

In this study, we emphasized the pressing need for a reevaluation of curricular tasks to include culturally responsive mathematics tasks and shared the collaborative efforts in revising the tasks for students, especially marginalized groups of students. Scholars have raised concerns that an ostensibly unbiased curriculum maintains a narrative rooted in white supremacy (Ladson-Billings, 2009a, b). This narrative can, in turn, intertwine students’ experiences with mathematics and their encounters with racialization and perpetuates systemic racism by marginalizing certain racial and ethnic communities (Gutiérrez, 2010/2013; Martin, 2006). Our efforts to change the curriculum have the potential to show students the relevance of mathematics in their lives, the value placed on their cultural practices and experiences within the school curriculum, and the role of mathematics as a means for enhancing their comprehension of human rights and agency.

In reviewing the 50 original mathematics tasks, we observed that these tasks consistently uphold high academic expectations. They necessitate a grasp of procedures intertwined with mathematical meanings, encourage the use of various representations, prompt students to discern their strategies, and provide opportunities for reasoning and justifying mathematical ideas (NCTM, 2014; Rubel, 2017; Smith et al., 2004). However, our examination revealed limited evidence of cultural competency and representation, and we did not identify any indication of sociopolitical and social justice issues embedded in the tasks. We endeavored to uphold or enhance the existing high academic expectations while incorporating other critical aspects of the Domains, such as c*ultural competency, social justice*, and *representation*. Throughout the revision process and reassessment of our modified tasks, we acknowledged the complexity of integrating social justice contexts into mathematical tasks. At various junctures, we deliberated on the potential discomfort or miscommunications that the added social justice context might introduce in the classroom. We recognized the challenge of striking a balance between embedding social justice content and maintaining the mathematical rigor present in the original tasks. Occasionally, these dual objectives limited the inclusion of the sociopolitical context we initially aimed to infuse, prompting us to reconsider the extent to which the social justice context was integrated.

The overview table resulting from this effort (Table 3) provided us with valuable insights as both researchers and teacher educators. It aids in the selection of tasks or lessons that focus on specific domains depending on the goals of the professional learning sessions, thereby informing the overall program design. Simultaneously, it identifies challenging domains for curriculum revisions, paving the way for continued exploration and iterative refinement in future research. We hope that the utilization of frameworks presented in Table 1, the evaluation process, and specific examples of both original and revised tasks would offer insights to researchers, teachers, and teacher educators interested in the evaluation, revision, and study of existing mathematical tasks to enhance cultural responsiveness for underserved communities.

In moving forward with the current socio-political turn in education, it is important to recognize that the work cannot rest on individuals alone. While teachers and teacher educators play a crucial role in sharing tasks with learners, meaningful and sustainable progress requires collective movement and professional learning—across grade-level teams, schools, districts, universities, and broader professional communities. In professional learning spaces, the discussion could focus on how this shift of tasks might influence instructional decision-making, the design and selection of tasks, and the need for pedagogical and broader support in safely facilitating socio-politically oriented mathematical discussions. This collective effort is especially important in the current political climate, where such work is challenging that narratives can quickly shift toward claiming the “neutrality” of the mathematics curriculum and where academic freedom is at risk. The work must be done carefully and intentionally, supporting teachers, educators, and researchers in navigating these challenges through sustained professional learning and discussion. Such support is especially important for those in more vulnerable situations, given the societal and political climates of their local contexts.

## Data Availability

Data and material used in this manuscript are available.
